# Bioactivity Profile of the Diterpene Isosteviol and its Derivatives

**DOI:** 10.3390/molecules24040678

**Published:** 2019-02-14

**Authors:** Asad Ullah, Sidra Munir, Yahia Mabkhot, Syed Lal Badshah

**Affiliations:** 1 Department of Chemistry, Islamia College University Peshawar, Peshawar 25120, Pakistan; asad_icp@yahoo.com (A.U.); saries92@gmail.com (S.M.); 2 Department of Pharmacy, King Khalid University, Abha 62529, Saudi Arabia

**Keywords:** stevioside, isosteviol, antitumor, diterpenoids, natural products

## Abstract

Steviosides, rebaudiosides and their analogues constitute a major class of naturally occurring biologically active diterpene compounds. The wide spectrum of pharmacological activity of this group of compounds has developed an interest among medicinal chemists to synthesize, purify, and analyze more selective and potent isosteviol derivatives. It has potential biological applications and improves the field of medicinal chemistry by designing novel drugs with the ability to cope against resistance developing diseases. The outstanding advancement in the design and synthesis of isosteviol and its derivative has proved its effectiveness and importance in the field of medicinal chemical research. The present review is an effort to integrate recently developed novel drugs syntheses from isosteviol and potentially active pharmacological importance of the isosteviol derivatives covering the recent advances.

## 1. Introduction

**Figure 1:**
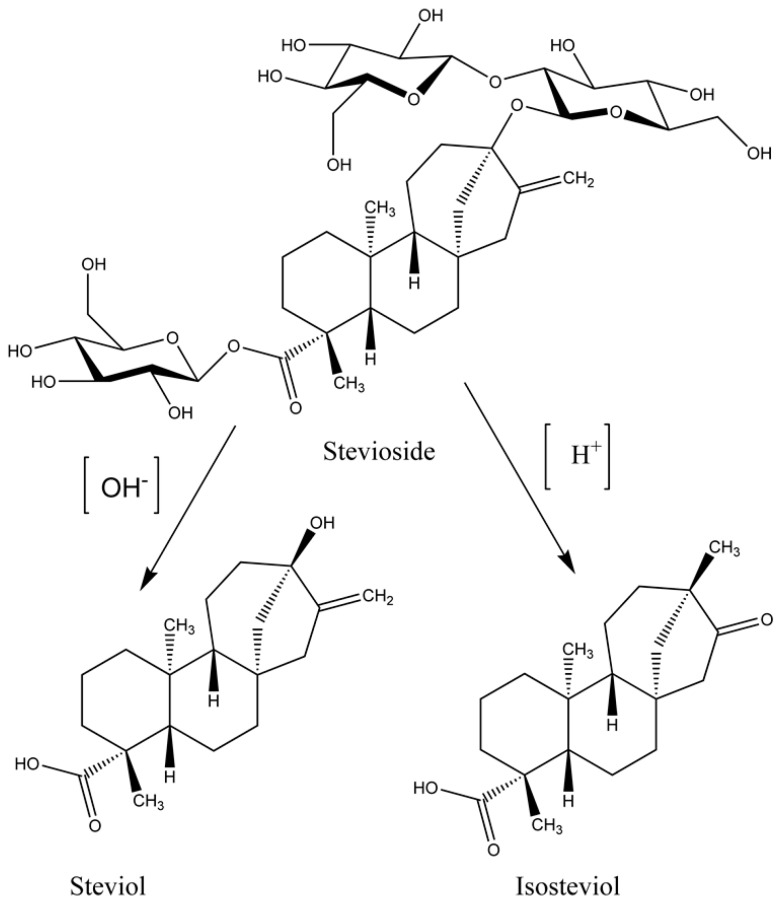
Formation of steviol and isosteviol from stevioside at different pH conditions.

The diterpenoid stevioside (Figure 1) is present in a significant amount in the leaves of the perennial herb Stevia (*Stevia rebaudiana* Bertoni) [1,2,3]. The stevia plant belongs to the chrysanthemum family of Asteraceae and it is native to the South American region. There are around 230 different species of Stevia but only two species named *S. rebaudiana* and *S. phlebophylla* producing the glycosides [4]. Among the huge number of available natural product compounds, the diterpenoids is a class of secondary metabolites that have gained remarkable attention in the pharmacological industry, not only because it exhibits a broad and diverse spectrum of biological activities, but also due to its easy availability. The diterpenoid glycoside, steviol glycoside (Figure 1) provides a promising alternative sweetener, which is non-nutritive, non-caloric [5], nontoxic, and non-mutagenic for patients having metabolic problems like Type II diabetes, phenylketonuria, and obesity [6]. There are no allergic reactions reported after its consumption [7]. Historically, Bertoni in 1905 reported the presence of a sweet constituent in Stevioside. Later in 1908, Deterich through hydrolysis method, isolated two glycosides, the non-crystalline rebaudin, and the crystalline eupatorine. The non-crystalline rebaudin was renamed as stevioside. The isosteviol (*ent*-16-oxobeyran-19-oic acid) is a sweet tetracyclic beyerene-type skeleton diterpenoid obtained through acid catalyzed hydrolysis of stevioside as first described by Bridel and Lavieille in 1931 [8]. The acid used is a strong mineral acid, such as, cold hydrobromic acid (48%) which gives isosteviol in good yield [9]. It cleaves off the glucose fragment from stevioside, because of the acidic environment the aglycone immediately rearranged from steviol to isosteviol (Figure 1). Apart from its sweetening properties, it exhibits several pharmacological activities [10] including anti-inflammation, anti-hypertension, controlling blood lipid, immunoregulation, antiviral, antibacterial, and a number of other medicinal and chemical properties [11,12]. It has been estimated that the total amount of the different steviol glycosides are from 4–20% of the total dry weight of the leaf [13]. In this percentage, the two major glycosides are stevioside and rebaudioside-A, where the total amount of stevioside is at a maximum of 7.9% and rebaudioside-A’s maximum yield is up to 6.5% (*w*/*w*) [14]. The rebaudioside is favored over the stevioside, as rebaudioside has high sweetness and tastes good [15]. The stevioside is 193-times sweeter than sucrose, while rebaudioside-A is 400-times sweeter. Further, rebaudioside M (Reb-M), which possess more β-glucose, is 200–350 times sweeter than sucrose and has a less bitter taste after consumption [16]. Due to the sweetness, its antidiabetic and anti-obesity role, several researchers have tried to exploit these properties and have patented a number of methods to increase the production, extraction, isolation, and purification of important steviosides and rebaudiosides, especially Reb-M [17,18]. The California based biotechnology company named Amyris and their partners are launching a zero-calorie sweetener that will contain Reb-M in 2019. They have claimed that their product is a fermentation-based product using sugar cane syrup and specialized microbes. There are other companies already in the market like PureCircle, which isolated stevioside based sweeteners from plants [19]. Thus, steviosides and rebaudiosides will be a big commercial product in the near future and will replace the table sugar commodity.

Increasing the production of glycosides in the plant is quite challenging and is an active area of research. Generally, the amount of steviosides are higher at the budding and flowering stage of the plant life [20]. It has been observed that long day period times before flowering increases the yield of glycosides. While other growing factors of water, nitrogen, and light conditions also affect the amount of glycosides in the plant [21,22]. Munz et al. tested two genotypes for the yield of stevioside and rebaudioside in southwestern Germany at field conditions in 2014–2015 at different light conditions [23]. They obtained a total steviol glycosides yield of 720–1023 kg/ha and rebaudioside A of 220–376 kg/ha in temperate conditions [23]. The generation of different genotypes that grow favorably in different climate conditions in the future will not only help in higher production of these important glycosides, but the cost will also be lowered. Li et al. utilizing a chemoproteomic profiling method found a steviol-catalyzing uridine diphosphate (UDP)-glycosyltransferase (UGT) UGT73E1 which is involved in the synthesis of steviol glycosides [24]. Yoneda et al. tested the effect of different light intensities and wavelengths on the expression of the steviol glycosides-related genes that are also part of the gibberellin’s biosynthesis pathway in the stevia plant [25]. They observed an increase in transcription of the UGT85C2 gene that translated into UDP-glycosyltransferases (UGTs) for catalysis of sugar-transfer reactions, and this gene was expressed more when blue, red, and far-red light emitting diodes were used as a light source [25]. Perera et al. have isolated several two new glycosides from the commercial leaf extract of *Stevia rebaudiana*, that include rebaudioside T, rebaudioside U, and prepared five steviol glycosides that have different sugar groups [26]. The attachment of variable monosaccharides at different positions on steviol may result in a stronger sweetener compound. After consumption of stevioside, the aglycone part steviol is separated in the gastrointestinal tract by the action of microbes and its enzymes. This free steviol is absorbed in the blood through the intestinal lining. Duesk et al. studied the interaction of the steviol with several receptor proteins of the liver that commonly interacts with different drugs and xenobiotics [27]. They observed that steviol interacts with pregnane X (PXR) and aryl hydrocarbon (AHR) receptors, while they also interacted weakly in vitro with two of the cytochrome P450 enzymes in the hepatocyte cells [27]. Thus, the stevioside and its metabolic products are safe for consumption and they have no harmful impact on the liver. However, further studies are needed for its effects on the blood–brain barrier and during pregnancy, on whether it is safe to consume or not. For commercial and large-scale production of plant based important secondary metabolites, the expression of the genes that codes for the enzymes involved in their metabolic pathway into yeast or the tobacco plant offered an opportunity [28,29]. Gold et al. expressed several genes that coded the different enzymes involved in the synthesis of steviosides in baker’s yeast, and the production of steviosides from glucose gave a good yield [30]. Thus, the commercial production of specific stevioside or rabaudioside can easily be obtained in large quantities through yeast fermentation.

Novel scaffolds have been synthesized by chemical modification of isosteviol core. Its synthetic and semisynthetic analogues possess potential therapeutic properties [31]. The different conjugated forms of isosteviol have anticancer activities and thus it can be used for the synthesis of inexpensive chemotherapeutic agents [32]. Being a metabolite of stevioside, it has wide biological activities and have also been approved by the European Food safety authorities and other regulatory bodies in different countries. Isosteviol also exhibits DNA polymerase and DNA topoisomerase inhibition properties, antibacterial, anticancer, acetylcholine inhibition [33], anti-diarrhea [12], antioxidant, and anti-tuberculosis effects. The goal of this article is to describe and outline the important nutritional, biochemical, and medicinal uses of isosteviol and its derivative compounds.

## 2. Chemistry of Stevia Glycosides

**Figure 2:**
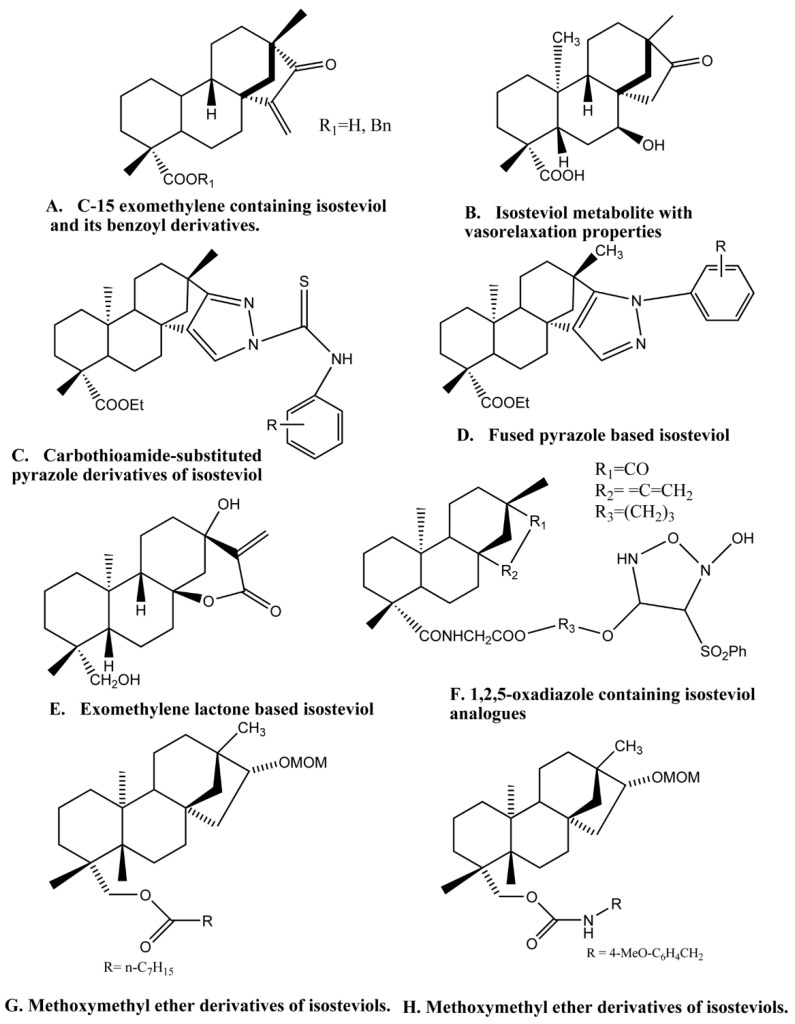
Bioactive derivatives of isosteviol.

The chemistry of stevia glycosides has caught the eye of researchers as the derivatives of it exhibit more significance than the parent compound itself, leading to the development of novel pharmacological active scaffolds. Furthermore, the unique and rare structural features of isosteviol make it a useful building block in organic synthesis. The stereochemistry revealed that the carboxylic and keto functional groups both digress from the parallel position by around 60°. This resulted in a lipophilic internal skeleton, while some hydrophilic polar moieties are on one side of the molecule. Such structures that are based on isosteviol, work as carriers and transport amino acids through fluid chloroform films [34]. Density functional theory calculations showed that the hydroxyl group on one side and the carboxyl group oxygen on the other side are the centers of the electron density localization and the hydrogen atoms are the most electropositive in its structure [35]. Calixarenes is one of the best examples of such carriers compounds that is enormously used in medicinal chemistry [34,36,37,38,39,40]. The synthesis of isosteviol based cages as a drug carrier needs to be explored as they are suitable carrier molecules. There are a limited number of reactive positions in the isosteviol molecule, therefore, chemical transformation is possible at only three positions. However, despite that, many derivatives of it can be synthesized employing a variety of methods. The most reactive moiety in the isosteviol molecule is the five membered D ring having a keto group which makes it a good target for chemical transformation. The transformation of isosteviol can be attained by different reagents under different conditions. For instance, stereoselective reduction of the keto functional group of C-15 exomethylene isosteviols with sodium borohydride in ethanol, yields the corresponding alcohols (Figure 2A) giving an R configuration product. The bromination of substrate 4 under basic condition leads to the product brominated alcohol. Similarly, treatment of isosteviol with sulfuryl chloride yield chlorinated product in high yield [41].

Bioactivity of isosteviol is similar to the gibberellin hormone and both share the same biosynthetic pathway [42]. The use of enzymes to carry out the chemical reaction is called biotransformation. Isosteviol undergoes biotransformation in the presence of the fungus. *Aspergillus niger*, *Penicillium chrysogenum*, and *Rhizopus arrhizus* are the three fungi used for biotransformation of isosteviol [43]. Biotransformation of isosteviol metabolites by *Mortierella isabellina* produce novel *ent*-beyeran-19-oic acids [44]. Maize infecting fungi, *Fusarium verticillioides*, is another fungus used for biotransformation producing *ent*-7,6-hydroxy-16-ketobeyeran-19-oic acid and *ent*-12-α-hydroxy-16-ketobeyeran-19-oic acid as their corresponding metabolites [45,46,47]. Fungal plant pathogen Gibberella fujikuroi undergoes isostevic acid biotransformation to give ring A desoxybeyer gibberellin analogues and hydroxylated beyrenes 7β-hydroxybeyeranolide [48,49]. These bio-transformations are performed to obtain metabolites that are more bioactive than the parent compound. *Cunninghamella echinulata* NRRL 1386 is the fungus used to obtain the isosteviol metabolite (Figure 2B) that has a 17-fold increase in vaso-relaxant activity as compared to the parent isosteviol [50]. Pancreatin catalyst demonstrated the best hydrolytic efficiency, producing isosteviol at a yield of 93.9% at low pH of 4.0 [51].

## 3. Plant Growth Regulator

A series of isosteviol derivatives such as aromatic esters and amino acid amides have been synthesized. Their inhibitory activity against seed germination and root elongation were studied. The gibberellic acid displays a role in germination of dormant seeds. Gibberellin and isosteviol are closely related in their activity and follow the same biosynthetic pathway, therefore, ester and amide substituted compounds of isosteviol derivatives were tested for their inhibitory activity against (*Capsicum annuum*); lentil (*Lens culinaris medicus*); tomato (*Lycopersicon esculentum*); clover (*Trifollium spp*.) and wheat (*Triticum vulgare*). The results showed excellent inhibitory activity in which a number of compounds synthesized were categorized into seed germination, root elongation inhibitors, root elongation inducers, and general inhibitors, that inhibit seed germination as well as root elongation. These classified biologically active groups widen the scope in which isosteviol can be used [52]. An isosteviol allyl ether was synthesized and its effect on winter wheat cultivar Mironovskaya 808 was studied [53]. The plant was cultivated in the laboratory for 12 h photoperiod with 100 W/m^2^ light intensity and a temperature of 23 °C. Isosteviol stimulated the leaf growth and delayed the root development at the optimum concentration of 10^−8^ M [53]. Isosteviol activates the secretion of α-amylase by the aleuronic layer in the course of seed germination [53]. This increases the productivity of the plant at a lower concentration. It also increases the activity of soluble lectins in non-hardened plants and a maximum increase in the leaf length [53]. All studied compounds induced an increase in the frost resistance of non-hardened plants but the most pronounced growth regulating effect was observed for isosteviol allyl ether [53].

Research into isosteviol and its derivative being used as a plant regulator, were carried out recently where the synthesized derivatives were tested on lettuce hypocotyl and barley aleurone bioassays. 17-hydroxy-16-ketobayeran-19-oic acid (17-hydroxyisosteviol, 6,16-ketobayeran-19-oic acid (isosteviol), 16,17-dihydroxybeyeran-19-oic acid, and 16-hydroxyiminobayeran-19-oic acid (isosteviol oxime) are the isosteviol moieties that were obtained by biotransformation of stevioside using *Penicillium citrinum* fungi [54]. The result of lettuce hypocotyl bioassay revealed that isosteviol was found to be more active than gibberellin acid at a concentration of <10^−9^ M of the activity, however, decreased at highest concentration [54]. The modification of isosteviol by the introduction of the hydroxyl group at C17, however, it resulted in the decrease in activity at a lower concentration [54]. The after effects of the barley aleurone bioassay demonstrated that compounds altogether prompted the development of an amylase [54]. In the beyerene skeleton, there was a critical increment in the reaction at the most minimal concentrations. The change of isosteviol was more powerful with its transformation to 16-hydroxyiminobayeran-19-oic (isosteviol oxime) [54]. Steviol and isosteviol additionally expanded the size and weight of berry affirming the bioassays signs of the potential utilization of steviol and subordinates as plant development regulators [54].

## 4. Pharmacological Activities of Isosteviol Derivatives

Isosteviol and its derivatives make up pharmacologically important drugs. Recent studies indicated their wide spectrum of medicinal and biological applications, which are discussed below.

### 4.1. Cytotoxic Agents

Cancer is the second driving reason for human mortality, surpassed by cardiovascular illness, and according to the International Agency for Research on Cancer (IARC), cancer is responsible for 9.6 million deaths and 18.1 million new cases recorded worldwide in 2018 [55,56,57,58]. Although several drugs are available, they do not yet meet the ever-growing need for drug resistant cancer cells. Discovery and development of efficient anticancer drugs are still essential and significant for mankind battling against persistent cancer cells [59,60]. Diterpenes are a natural product-based compound, which are a rich source of potential therapeutics [61]. Paclitaxel or taxol is probably the most well-known diterpene based anticancer agent [62,63]. Recently stevioside based diterpenes, isosteviol, and its derivatives have gained attention. They act as a natural chemotherapeutic agent and can be transformed by both microbial change and synthetic adjustment. Chemical change of one or more reactive positions of isosteviol leads to the formation of hundreds of potentially potent novel scaffolds exhibiting cytotoxic activity. For example, four different scaffolds of tetracyclic diterpenoids were prepared from isosteviol and steviol. A compound with exo-methylene cyclopentanone structure was synthesized from isosteviol displayed significant cytotoxic activity against several human cancer cell lines and some have half the inhibitory concentration (IC_50_) values from 0.09–5.71 μM, and most of the derivatives have superior cytotoxicity against the different cancer cell lines than in the positive control doxorubicin [64]. Quantitative structure activity relationship (QSAR) models are used to reveal physiochemical properties, structural descriptions (such as connectivity and topological) etc. [65]. Several cytotoxic activities of tested cancer cell lines can provide a guideline for the synthesis of novel and more potent anticancer agents [66]. Isosteviol derivatives are α-glucosidase inhibitors. The glucosidases enzymes are highly focused on malignancy and other hereditary disorders. A series of isosteviol (*ent*-16-ketobeyeran-19-oic acid) derivatives were quantitatively studied by QSAR. Results indicated that some subunits of isosteviol derivatives inhibit the growth of cancer cells [67].

The amino alcohol exhibits a wide range of biological activities and is part of the structural fragments in synthetic molecules and natural products [68]. Amino alcohol in the D-ring of isosteviol was developed and a series of its analogues were acquired with essentially enhanced anticancer activity [69]. Another promising class of anticancer agent is the thiourea derivatives that possess significant inhibitory action against topoisomerase II, protein tyrosine kinases, and human sirtuin type proteins 1 and 2 [70,71]. The cytotoxic activity of isosteviol increases when isosteviol analogues have amino alcohol and thiourea groups and it was observed that they are active against three human cancer cell lines. Each of the derivatives of isosteviol discovered showed preferable cytotoxic activity over their precursor compound [72].

The D-ring modification of isosteviol could change its natural activity or prompt new action. A modified D-ring with fused heterocyclic analogs further improves the cytotoxic activity. Below are a few examples. Carbothioamide-substituted pyrazole and isoxazolidine are important building blocks of many pharmacologically active compounds [73,74,75,76]. Both fragments display enhanced biological activity upon introduction to another compound [77,78,79]. Two series of carbothioamide-substituted isoxazolidine isosteviol analogues containing pyrazole and isoxazolidine ring fused with the isosteviol structure on integration exhibited novel antitumor activity [73]. These bioactive molecules showed promising results when tested against four human tumor cell lines: Gastric cancer (SGC 7901), lung cancer (A549), lymph cancer (Raji), and cervical cancer (HeLa) [73]. The carbothioamide-substituted pyrazole derivatives of isosteviol (Figure 2C), are the most potently cytotoxic compound against Raji cell lines with an IC_50_ value of 6.51 μM [73].

D-ring modification of tetracyclic diterpene isosteviol is exemplified by 15- and 16-substituted isosteviol derivatives that were selectively prepared and contain a pyrazoline and isoxazolidine ring fused with an isosteviol structure. Some of these compounds display promising cytotoxic activities and were tested against B16-F10 melanoma cells [80]. Furthermore, isosteviols fused with heterocyclic compounds resulted into novel therapeutic agents such as Isosteviol-fused pyrazolines and pyrazoles, that are potential anticancer agents. Two novel series of these isosteviol derivatives were also stereoselectivity synthesized and their test against four human cancer cells are noteworthy. The isosteviol-fused pyrazole compound (Figure 2D) showed the strongest cytotoxicity, which can be used as a lead compound for the advancement of potent antitumor agents [81].

When a methylene lactone group was introduced into the isosteviol through structural modification, it produced three scaffolds of *ent*-kaurene diterpenoids and their anticancer activity were tested against six human cancer cell lines [64]. One of the compounds, which was methylene lactone isosteviol analogue (Figure 2E), showed the most activity against HepG2 cell lines with an IC_50_ value of 9l mM [82,83]. The functionalization of isosteviol using hydrazone moiety has proved to be effective biologically [84]. The 2,4-dinitro phenyl hydrazone (2,4-DNPH), 4-nitro phenyl hydrazone (4-NPH) are two such derivatives that have been synthesized and tested for their cytotoxic, antimalarial and anti-trypanosomal and anti-leishmaniasis activities. The results indicated that the derivatives were moderately to highly active.

The nitric oxide (NO) donating drugs are efficiently developed nowadays, the most successful of which is NO-nonsteroidal anti-inflammatory drugs (NO-NSAIDs). In one such study, fifteen novel compounds have been synthesized, where the nitric oxide (NO) donor is incorporated into the isosteviol structure for an anticancer purpose. These compounds were tested against a hepatocellular liver carcinoma cell line (HepG2) and highly metastatic melanoma cells (B16F10). The oxadiazole ring containing isosteviol derivative (Figure 2F) with an IC_50_ of 0.02 μM has the strongest effect and is the lead compound for a further therapeutic investigation [85]. Twelve isosteviol derivatives were synthesized and tested against hepatocellular carcinoma (HCC) cells for evaluation of anticancer properties in the liver. Isosteviol analogues number 10-C with IC_50_ of 2 μM, inhibited apoptosis in HepG2 cells. Different observations showed that these derivatives encouraged apoptosis in HepG2 cells, blocked angiogenic signaling and it didn’t cause toxicity in the treated hosts [85]. Another researcher used the same approach, synthesizing twenty-six novel isosteviol derivatives coupled with two types of nitric oxide (NO) donors (furoxans and NONOates) [86]. Seven of the furoxan based derivatives were tested for their in vitro cytotoxic activity against four cancer cell lines HCT116, Huh7, HepG2, and SW620, while the NONOates displayed negative result [86]. These derivatives can be lead compounds for further research. Greater than the amount of NO released by compounds enhances its activity [85,86]. Some of the derivatives of isosteviol with nitric oxide releasing properties have a higher potency than the comptothecin [85]. Acylation of the 19-OH group of kaurane- and beyerane-type diterpenoids may be helpful for the upgrade of their cytotoxicity with apoptosis inciting action [87]. Although it is difficult to synthesize isosteviol derivatives that are specifically cytotoxic to target cells without harming the normal cells [88]. In this regard, 19 isosteviol derivatives were synthesized and tested against four different carcinoma human cell lines, among which five isosteviol derivatives exhibited activity against one or more cell lines. One of the most common malignancies in the world is lung cancer. Novel and highly selective MOM-ether analogs of isosteviol (Figure 2G,H) were designed and synthesized, and tested against H1299 lung cancer cell lines [89]. Out of 12 derivatives, two displayed potent activity in reducing cytotoxic effects on NL-20 normal lung epithelial cells. These MOM ether derivatives have IC_50_ values of 14 and 21 μM against lung cancer cell lines [89]. The ether analogs of isosteviol will prompt the improvement of novel anticancer medications. However, more work is needed in this area to explore these natural bounties [90]. Lin et al. developed isosteviol derivatives that were evaluated for cytotoxic effects against growth inhibition of three cancer cell lines and human embryonic lung cells MRC-5. The results were significant and provided a convincible anticancer agent [90]. Some isosteviols are presented naturally and can be an excellent anticancer agent. For instance, roots of *Ceriopsins decandra* have two beyerene ceriopsin G and isosteviol [91,92]. Alijani et al. made zinc sulfide (ZnS) nanoparticles of stevia extract, which are stabilized by the glucose moiety [93]. These nanoparticles were active against the human cancer cell line MCF-7 with an IC_50_ value of 400 μg ml^−1^ [93]. For over 50 years, the main goal of researchers in cancer therapeutics is to design and synthesize such universally effective drugs that are not only an inhibitor of tumor cells but have less toxicity for normal cells. The use of isosteviol as a natural chemotherapeutic agent is gaining interest among the scientific community and it could be a possible solution for controlling different types of cancers.

### 4.2. DNA Polymerase and DNA Topoisomerase Inhibitors

DNA polymerases are enzymes that assemble nucleotides to create DNA. These enzymes are fundamental to DNA replication, and for the most part, work in sets makes two DNA strands from one unique DNA molecule. DNA is subjected to damage from many sources and repairs of these damages by different pathways relied on a polymerase enzyme, therefore, making it a crucial element by which cancer cells can endure DNA harm. Some of these enzymes are suitable for helpful chemical biology techniques [94]. DNA topoisomerases are those enzymes that manage the topological state of the DNA in the cell [95,96,97,98]. These include steady state alteration of supercoiling, DNA replication, transcription, recombination, and chromatin remodeling [95,96]. Topoisomerases in both bacteria and humans are the target of many drugs [99,100,101]. These drugs mostly generate cytotoxic lesions by trapping the enzymes in covalent complexes on the DNA [95,96,97,98]. It was also observed that isosteviol is active against these two enzymes, especially 1,2,5-oxadiazole containing isosteviol analogues (Figure 2F), which is a potent inhibitor of both mammalian polymerase (α, β and ∆) and human DNA topoisomerase II [102]. It also halts the growth of human cancer cells and 12-*O*-tetradecanoylphorbol-13-acetate (TPA) induced inflammation [102]. The isosteviol analogues also inhibit the activity of mammalian polymerase especially pol-α by interacting with the enzyme directly [102].

### 4.3. Antiviral Agents

Scientists have always been in search of selective and potent anticancer and antiviral drugs to fight the continuously increasing drug resistant health problems. Chronic infection of the liver and hepatic cell carcinoma (HCC) is also related to the Hepatitis B virus (HBV) [103]. As stated by WHO estimates, there are about 5 million cases of acute hepatitis B infection each year [104,105] and 0.6 million patients die each year from HBV-related liver disease [106]. Despite a large number of recombinant vaccines available, the need for the specific HBV immunoglobulin drug against frequent new infections is of the utmost necessary [107]. In this regard, interferon (IN)-a and nucleoside derivatives were developed [108]. Yet the low-to-moderate efficacy, unwanted side-effects, and drug resistant viral strains make this problem worse [109]. Considering these continuing efforts for the synthesis of new antiviral agents with novel targets and mechanisms to eradicate HBV. Several isosteviol derivatives were prepared by replacing the 19-COOH with the ureide moiety, and their inhibitory action against HBV was evaluated [109]. Among them, NC-8 *ent*-16-oxobeyeran 19-N-methylureido, showed inhibitory activity against HBV and specifically inhibited viral gene expression and reduced the level of encapsulated viral DNA intermediates in Huh7 cells that expressed the replicating HBV [110].

**Figure 3:**
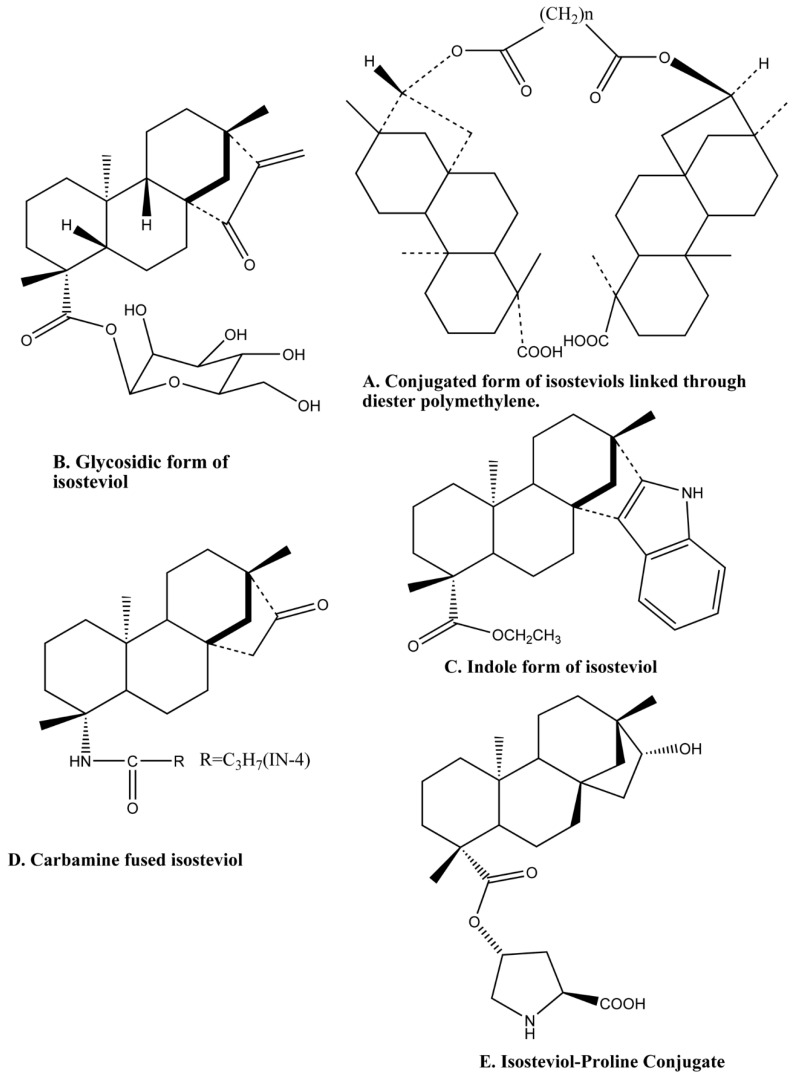
Different analogues of isosteviol.

In-vitro synthesis of the isosteviol derivative having C4-amide moiety is designed as an antiviral HBV agent. Among them, IN-4 [*N*-(propyl carbonyl)-4-amino-19-nor-*ent*-16-ketobeyeran] (Figure 3D) displayed inhibition of not only HBV DNA replication but also that of secretion of HBsAg and HBeAg [111]. It significantly inhibits HBV gene regulation by disrupting nuclear factor (NF)-B-associated promoter activity that halts viral gene expression and DNA replication [111]. The stevioside is the raw material for the synthesis of (−)-Tripterifordin and (−)-Neotripterifordin that are potential inhibitors of the HIV replication process [112]. These two compounds can be made in 9 to 11 steps with 5 to 7 isolation steps in this synthesis process from steviosides [112]. The tripterifordin has an IC_50_ of 3100 nM and neotripterifordin has 25 nM against HIV replication in H9 lymphocyte cells [112].

#### Early Antigen Activation of Epstein–Barr Virus (EBV-EA)

A new strategy is proposed to control the development of cancer by chemoprevention using natural products such as fruits, vegetables, and pharmacological agents with a wide range of activity against different cancer types [113,114,115,116,117]. There has been extensive research on using diterpenoids and triterpenoids for inhibition of the Epstein–Barr virus early antigen (EBV-EA) activation induced by tumor promoter 12-*O*-tetradecanoylphorbol-13-acetate (TPA) [11]. Recently, the inhibitory effects of the natural sweetener stevioside and its aglycone isosteviol have been reported on EBV-EA activation. Steviol and Isosteviol both exhibited strong inhibitory effects in a two-stage carcinogenesis test using mouse skin induced by the initiator 7,12-dimethylbenz[a]anthracene (DMBA) or peroxynitrite and promoter TPA. These results suggested that steviol and isosteviol exhibited greater inhibition than glycyrrhizin and comparable activity as that of curcumin and, therefore, can be used as a natural chemo-preventive agent for carcinogenesis [11]. Teleocidin isolated from Streptomyces mediocidicus is another type of potential promoter with potencies equivalent to those of TPA. One of the reports suggests that stevioside also might be as potent as TPA in term of anti-tumor-promoting activity on mouse skin against teleocidin. This is the first finding to prove that stevioside may protect tumor promoting potency from microbiological products [41,118]. Thus, the microbial transformation is a significant technique for structurally modifying natural and synthetic compounds due to its significant regio- and stereoselectivities [119,120,121,122]. Fungi are widely used in microbial transformation studies since their versatile enzymatic reservoir allows them to modify a diverse array of molecules [119,120,121]. Microbial transformation of isosteviol by fungi yields 5 metabolites 7*α*-hydroxyisosteviol, 11*α*-hydroxyisosteviol, 12*α*-hydroxyisosteviol, 17-hydroxyisosteviol, and 7-oxoisosteviol [119,120,121,122]. These compounds were evaluated for their inhibitory effects on EBV-EA promoted by TPA. All these compounds exhibited a more potent inhibitory effect than the parent isosteviol [123].

### 4.4. Antibacterial Agents

Gram negative and gram-positive bacteria are responsible for bacterial infection affecting millions of people every year. These pathogens are developing resistance against newly synthesized drugs and therefore the demand for novel effective drugs is increasing day by day [101,124,125]. A series of novel compounds were synthesized by different methods that contain indole, pyrazoline, and isoxazolidine rings fused with the isosteviol framework [126]. These compounds showed antibacterial properties. Isosteviol derivatives that are mono and diesters of isosteviol, containing onium nitrogen atoms were synthesized and tested for their antimicrobial and antifungal activities. Two derivatives of isosteviol were formed during the process and both were obtained by the reaction of isosteviol with thionyl chloride in excess producing acid chloride [34], the resulting compound reacting with *N*,*N*-dimethylaminoethanol in carbon tetrachloride (CCl_4_) producing a mixture of products. Chromatographic extraction followed by the H-NMR spectrum of the resulting compounds revealed its characteristic mono and diester structures. The obtained di and mono esters displayed efficient antibacterial properties [127]. Ammonium derivatives of the diterpenoid have also been synthesized. The compound having a dodecamethylene spacer between two quarternized nitrogen atoms had the greatest antimicrobial activity. The greater the number of quarternized nitrogen atoms in an ammonium compound, the greater their biological activity. Many compounds were synthesized using the same concept, in which several compounds displayed good antibacterial activity [128].

#### Anti-Tuberculosis Agents

The use of conjugation of two or more compounds has increased recently for potency and better solubility. They are either placed in a large carrier molecule through non-covalent interaction or through covalent conjugation of the known drugs to other biologically active compounds and cell-penetrating carrier compounds [39,40]. The same approach is used when conjugates of diterpenoid isosteviol and drug dimephosphon (1,1-dimethyl-3-oxobutylphosphonic acid dimethyl ester) are synthesized [129,130]. The developed drug has increased the tuberculostatic activity by ten-times more than isosteviol. It effectively inhibited the growth of *Mycobacterium tuberculosis* H37Rv in in vitro conditions [129,130].

Isosteviol exhibits moderate antitubercular activity. When two isosteviol molecules are linked by a polymethylene spacer with C16 atoms and increase the number of methylenes in the spacer from one to eight, it decreases the minimal inhibitory concentration. Hence, it is also found that functionalization of N-containing groups (hydrazide and hydrazone) into isosteviol and its bis-derivatives increase their antitubercular activity and reduce the minimal inhibitory concentrations (MIC) [129,131,132]. Furthermore, hydrazone subsidiaries and conjugates holding two isosteviol moieties with a dihydrazide linker were acquired. Those parental compounds and their manufactured subsidiaries restrain the in vitro growth of *M. tuberculosis* (H37RV). The maximal inhibitory effect against *M. tuberculosis* was shown by the isosteviol conjugates with adipic acid dihydrazide (MIC 1.7 and 3.1 μg/mL) [133]. Isosteviol is a well-known growth regulator with a tendency to perform the oxidative phosphorylation shell of *M. tuberculosis*. On this basis, a series of bis(isosteviol) alkanones were synthesized for anti-tuberculosis activity. Functionalization of isosteviol is done by linking a diester polymethylene spacer to another *ent*-beyeran fragment at the C-16 atom (Figure 3A). The resulting diesters 15–18 of the parent molecule were obtained with 30–35% yields. These compounds were evaluated for their anti-tuberculosis activity and it was established that the synthesized derivatives exhibited direct anti-tuberculosis action, which relies on the length of the polymethylene spacer. The carbamine fused isosteviol analogues (Figure 3D) had a MIC value of 12.5 μg/mL. Hence, new anti-tuberculosis drugs among isosteviol can be made by functionalization of isosteviol and increase in the methylene spacer atom [134]. A macrocycle has been synthesized which contain twos isosteviol and a malonate moiety. This conjugated macrocycle has been tested for its capacity to control the development of *M. tuberculosis* H37Rv in vitro and showed a MIC estimation of 1 mg cm^−3^. The macrocycle incorporates fullerene C_60_, thus an adduct of this macrocycle and fullerene is established and it is called methanofullerene. This conjugated form of the molecules is active against tuberculosis [132,135].

The synthesis of the first macrocyclic glycoterpenoid having isosteviol and glucosamine residues were also made. The terminal reactive groups of the binuclear isosteviol derivative were functionalized with carbohydrate residues, and they were then coupled by the linker. The diol-1 was used as a starting compound in which two isosteviol molecules were bonded to each other with an octamethylene linker attached to their C-16 atoms, while carboxyl groups of these isosteviol moieties were functionalized by 6-hydroxyhexyl chains. It was found that this compound inhibited the in vitro growth of this strain of pathogen at a MIC of 12.5 μg/mL, that is, at the level comparable to the known antitubercular drug pyrazine amide. In conclusion, the macrocyclic glycoterpenoid having two isosteviol moieties and two glucosamine residues bonded by polymethylene linkers with ester and amide groups has been synthesized for the first time (Figure 3A). It inhibited the in vitro growth of the H37Rv strain of *M. tuberculosis* at the MIC value of 12.5 μg/mL.

Another researcher developed macrocyclic glycoterpenoids, a new class of biologically active compounds, containing diterpenoid isosteviol and glucuronic acid fragments. The bioavailability of the newly synthesized substances is increased by glucuronic acid, which transforms hydrophobic substances into hydrophilic substances. For this reason, the macrocyclic analogues of glucuronic acid derivatives containing several isosteviol fragments have been synthesized. The obtained macrocycle exhibits anti-tuberculosis activity against H37RV, *M. avium*, and *M. terrae* strains [88].

Naturally occurring macrocyclic glycoterpenoids have a wide variety of applications. Conjugating glucosamine to biologically active compounds significantly reduces its hepatotoxic and immunotoxic effects. Recently, the first synthesis of macrocyclic glycoterpenoids including glucosamine and isosteviol moieties have been carried out, and their ability to inhibit *M. tuberculosis* H37Rv in vitro was successfully evaluated. The synthesized compounds showed moderate tuberculostatic activity with low a MIC value of 6.4–17.4 μM [136]. Anti-tuberculosis activity is related to the structure and geometry of the tetracyclic diterpenoid skeleton and this is showed by the new semisynthetic *ent*-kauranes. 16(*S*)-dihydrosteviol, which has been synthesized and is used for obtaining *ent*-kaurane analogues of unfolded and macrocyclic derivatives of isosteviol exhibiting tuberculostatic activity. It was found that the shorter diester linker results in greater anti-tuberculosis activity [137].

### 4.5. Antihypertensive Agent and Cardio Protection

Hypertension is the condition where blood pressure is 140/90 mm Hg and the patient is taking antihypertensive medications for its control. It is one of the major causes of cardiovascular diseases including myocardial infarction, stroke, heart failure, and renal failure etc., affecting over 65 million adult Americans [138,139]. Diuretics and beta blocker drugs like propranolol beta-adrenoreceptor drugs [140,141], or Nifedipine calcium channel blockers, are the most commonly used antihypertensive drugs [142]. However, these drugs have a negative impact on the quality of life and show toxic side effects [143,144] such as amnesia, lethargy, coma [145], or even death of an individual occurs when consumed in high dose [146,147]. Thus, the need for a natural product to lower blood pressure that has a less toxic effect has been needed. Isosteviol has been reported to reduce vasoconstriction and regulate blood pressure. One of the studies used aortic rings isolated from Wistar rats, where isosteviol acted on the potassium channel [148]. There are five subtypes of potassium channel [149,150]. Among them, ATP-sensitive potassium (KATP) channels may be the cause of temporarily activated potassium channels and permits impact for intracellular Ca^2+^ concentrations [148,151]. Blockers are needed for each subtype of potassium channel to evaluate the activity of isosteviol [148,151]. The vasodilator impact from claiming isosteviol might have been lessened markedly in the vicinity for glibenclamide blocker demonstrating this reaction will be interceded through a KATP channel [151]. Those could reasonably be an expected mechanism for bringing down intracellular Ca^2+^ concentration, eventually by isosteviol, in addition to opening the potassium channel [151]. The mechanism for aortic relaxation through isosteviol might be helpful in the synthesis of novel vasodilator substances. Isosteviol dose-dependently loosens vasopressin prompted vasoconstriction through separated aortic rings with or without endothelium [151]. However, in the presence of potassium chloride, those vasodilator impacts from claiming isosteviol considering blood vessel strips disappeared. It has been suggested that vasodilatation prompted by isosteviol will be identified with the opening for different ions and K-ATP channels [151]. Another study showing the antihypertensive effect of isosteviol through calcium influx inhibition in vascular smooth muscle cells when intraperitoneal injection into spontaneously hypertensive rats was administered [152]. Using Guinea pigs as an animal model to prove the cardioprotective properties of isosteviol against myocardial ischaemia–reperfusion (IR) injury [153], pigs were divided into seven different groups and a control group [153]. Guinea pig hearts were isolated and ischemia followed by reperfusion was performed. Many activities along with cardiac function were monitored [153]. The perfusion control group displayed no significant change in the cardiac function, however, pretreatment with isosteviol (in 50, 250, or 500 nmol concentration) had a significant decrease in cardiac function with *p* < 0.05. Isosteviol didn’t build coronary flow, suggesting that the protective impact of isosteviol on the myocardium might have been not interceded by expansion of the coronary blood vessels [153]. Pretreatment with those mito-KATP blocker 5-HD incompletely antagonized the impacts of 500 nmol isosteviol [153].

### 4.6. Neuroprotective Effect

Impairment of blood flow to the cerebral artery leads to the death of neurons. Stroke, necrosis and other damage to the brain are associated with such impairments. Isosteviol has been accounted for a defensive impact in damage to the cerebrum. An experiment was performed in focal cerebral ischemia injury induced by middle cerebral artery occlusion in rats. The outcome demonstrated the pretreatment with isosteviol alleviated ischemia-reperfusion (IR) damage in vitro. It also reduces cell death, inflammation, and infarct volume of the myocardium, which is increased by opening and re-opening the coronary artery in the rat in vivo [154]. An isosteviol sodium injection was administered to rats having either permanent or transient middle cerebral artery occlusion to study the neuroprotective effect of isosteviol in rats. The infarct volume was considerably reduced and the isosteviol sodium (STVNa) treatment suggests largely reduced neurobehavioral impairment proving to be a potential therapeutic agent against cerebral ischemia-induced injury [155].

### 4.7. Antagonists of Angiotensin II

The antagonists of angiotensin II are vasoconstrictor agents, which are therapeutically used as antihypertensive drugs or in heart failure conditions. A series of isosteviol derivatives, which are considered as antagonists of angiotensin II, were tested on rat aortic smooth muscle cells. These derivatives indicated that isosteviol inhibit angiotensin-II-induced cell proliferation and endothelin-1 secretion, reactive oxygen species generation, suppresses extracellular signal-regulated kinase (ERK) and phosphorylation in vascular smooth muscle cells [156,157].

The antioxidative impact of isosteviol on angiotensin-II related reactive oxygen species generation in hypertensive injury of aortic smooth muscle cells was also observed. Isosteviol controls the production of ROS, which is formed in Ang-II induced proliferation. Cultured rat aortic smooth muscle cells were preincubated with isosteviol and then stimulated with angiotensin II, after which [3H]-thymidine incorporation and endothelin-1 secretion were analyzed. Isosteviol inhibits angiotensin-II-induced DNA synthesis and endothelin-1 secretion. Isosteviol-intervened inhibition of intracellular reactive oxygen species (ROS) created by the impacts of angiotensin II was shown by the estimation of redox sensitive florescent dye. The inductive properties of angiotensin II on ERK phosphorylation were discovered to be turned on with isosteviol and antioxidants, for example, *N*-acetyl-cysteine [158,159].

### 4.8. Anti-Inflammatory Activity

Inflammation is a complex pathophysiological and dynamic process involving multiple cellular and molecular interactions, which are mediated by activating inflammatory or immune cells [160,161]. Biotransformation of isostevic acid resulted in the formation of fourteen oxygenated compounds, which upon incubation formed sixteen metabolites. There were fifteen that exhibited significant in vitro anti-inflammatory activity in lipopolysaccharide (LPS)-stimulated RAW264.7 macrophages by reducing the levels of both TNF-α and COX-2 mRNA relative to the control cells stimulated by LPS alone [160]. Although liver cirrhosis is commonly caused by viral infections and alcohol over-consumption, oxidative stress plays a significant role in this disease [162]. The nuclear erythroid factor 2 (Nrf2) regulates the redox process of the cells, and it has been observed that it becomes dysfunctional in cirrhosis [162]. The use of steviosides in murine models upregulates the Nrf2 and controls the oxidative stress by ceasing inflammation through inhibition of the nuclear factor factor-κB (NF-κB) in carbon tetrachloride (CCl_4_) induced liver cirrhosis [162]. In another similar study, it was also observed that the consumption of aqueous extract from *Stevia* in rats heals the fibrotic liver [163]. The stevioside and other related glycosides present in the *Stevia* extract block profibrogenic signaling pathways and proinflammatory cytokine production, and through this way they inhibit the fibrosis of the liver [163].

### 4.9. Anti-Hyperglycemia Effect

#### 4.9.1. Glucose Receptor Sensitization

Hyperglycemia is the abnormally high glucose level condition. Some studies suggested a decrease in glucose levels in the diabetic animal model to prove the anti-hyperglycemic effect of isosteviol. Isosteviol tends to decrease blood glucose concentration in the intravenous glucose tolerance test in Zucker diabetic fatty rats. This decrease by isosteviol is associated with changes in the sensitivity of peripheral tissues to insulin. The result was, however, negative for Wistar rats [164]. The induction of hyperglycemia in rats by daily injection of high fat emulsion is evaluated with oral administration of the isosteviol dose. Results indicated anti-hyperglycemic impacts of isosteviol could improve the use of glucose in the periphery and diminish β-cell harm initiated by dyslipidemia. Balancing lipidemic impacts of isosteviol may be identified with the potential improvement of liver PPARα mRNA [165].

Another test confirming the decrease of plasma glucose level in Zucker diabetic fatty (ZDF) rats was performed by Ma et al. The intravenous glucose tolerance test (IVGTT) was performed on normal Wister rats and ZDF rats, which were divided into control and test groups. The rats were fasted for 12 h before infusion of isosteviol and glucose. Blood samples were taken at different time intervals and the glucose concentration for each interval was examined using the glucose oxidase method and plasma insulin concentration was determined by radioimmunoassay. The result of the control and test groups were then compared. Isosteviol had no effect on the glucose concentration of the normal Wistar rat, however, in the case of ZDF rats, the plasma glucose level decreased significantly. The β-cells of pancreas undergo changes with time to develop insulin resistance and ultimately Type-II diabetes. An obese mouse suffering from Type-II diabetes and KKAy mice (Kyoji Kondo [KK] diabetic genes containing mouse and Ay for yellow obese gene, that is mouse with both diabetic and obese genes) were used as a model to understand the progression of diabetes. For the KKAy mice, it took 12 weeks to reach the insulin deficient stage. Isosteviol builds the glucose affectability and changes the quality profile of basic insulin administrative qualities and β-cell transcription factors in the secluded islets after a long-haul in vivo mediation. At week 9 of the experiment, there was a 266-fold increase in the plasma insulin for the KKAy control group. At the end of the study, this increase was 2.8-fold for KKAy control group while there was a 1.8-fold increase for the KKAy treatment with isosteviol. Glucose–insulin index for the isosteviol (ISV) group 6.9 compared with the KKAy control 1.6 illustrating that ISV has decreased the insulin resistance [166].

Along with β-cells, there are other cells such as α, δ, γ, ε. The α-cells are glucagon-producing endocrine cells, which reside in the Islets of Langerhans. The main role of glucagon in diabetes is that its decreased secretion leads to a hyperglycemic state in Type-II diabetes. Patients with Type-II diabetes are associated with a high level of fatty acid concentration, which influences glycogen secretion. The effect of isosteviol on Type-II diabetes was investigated by taking α-TC1–6. The α-cells were cultured for 72 h with palmitate concentration of 0.5 mM and 18 mM glucose. An increase of 56% and 78% in glycogen and triglyceride content of α- cells were observed respectively. Using the same concentrations and repeating the procedure with the introduction of isosteviol having a concentration of 10^−8^ and 10^−6^ M, both of which reduced palmitate-stimulated glucagon release by 27% but neither of these had any effect on the triglyceride content of α-cells in the presence of palmitate. However, it upregulated the gene expression near palmitate. The isosteviol neutralizes α-cell hypersecretion and furthermore adds to changes in expression of key genes coming about because of long time exposure to palmitate. These results showed that isosteviol may possibly become a glucagonostatic drug with potential as diabetic medication for the treatment of Type-II diabetes [3].

A total number of plasma metabolites in KKAy mice suffering from Type-II diabetes was given isosteviol, an anti-diabetic diet for 9 weeks as well as a high isoflavone soy diet protein for 9 weeks [167]. NMR spectroscopy was used to study the chemical features of metabolites of normal C57BL mice and control KKAy mice. The KKAy mice treated with soy protein aligned with the group of KKAy control mice, while the KKAy mice treated with ISV mainly aligned together with the C57BL group [167]. The difference between the two groups was because of the difference in lipoprotein composition [167]. The abnormalities are in plasma triglycerides and plasma lipoproteins [167]. The Type-II diabetic mice treated with isosteviol had a clear change in plasma composition of metabolites as compared to one left untreated [167]. The soy protein diet also influences lipoprotein, but it was to a lower extent than that of isosteviol [167]. Isosteviol has low absorption in the human body in order to increase the bioavailability of the analogues of the said compound were synthesized, which displayed a better performance. In this regard, isosteviol was converted via many reactions into 1,2,4-triazine [59,168,169]. The diabetes mellitus also resulted in hyperglycemia, generation of reactive oxygen species (ROS) and also affecting skeletal muscles [170]. It has been observed that consumption of steviosides enhances the activity of AMP-kinase and GLUT4 (an insulin dependent glucose transporter in muscles) that resulted in strengthening of muscles and inhibition of ROS generation [171].

#### 4.9.2. α-Glucosidase Inhibitor

Inhibitors of α-glucosidase of the small intestine caused an interruption in the digestion of complex carbohydrates by acting as a competitive inhibitor. Thus, they prevent the conversion of oligosaccharides into simple monosaccharides [172,173]. Carbohydrate mediated diseases such as diabetes and obesity etc. are treated using α-glucosidase inhibitors [172]. A series of glycosylated derivatives of tetracyclic diterpenoids containing an *exo*-methylene cyclopentanone or an *α*-methylene lactone moiety were synthesized, and their cytotoxic activities were examined. Two of the scaffolds were synthesized from isosteviol by 10% acidic hydrolysis of H_2_SO_4_. The cytotoxic effects of these compounds were tested against six different cancer cell lines, these include human hepatocellular carcinoma cell lines (HepG2, Bel-7402), human lung cancer cell line (A549), human glioma cell line (U251), and breast carcinoma cell lines (MCF-7, MDA-MB-231). The glycosidic form of isosteviol (Figure 3B) and its analogues exhibited selective inhibition of some of the tested cancer cell lines [83]. Another expanded series of compounds containing hydroxyl, a hydroxymethyl group and heteroatom-containing frameworks fused with the isosteviol structure were synthesized and was found to inhibit α-glucosidase with moderate to good activity. The next lead compound that was further investigated was the indole derivative (Figure 3C) with the highest activity [174].

## 5. Other Miscellaneous Uses

### 5.1. Chiral Catalyst

The search for effective catalysts has always been important. l-proline is an important amino acid that acts as a catalyst, and is used for the direct asymmetric aldol reaction, however, it scatters in water and therefore gives an unsatisfactory result in reactions occurring in aqueous media. Two derivatives of proline were synthesized by condensation of l-proline with isosteviol in a one-pot synthesis. The obtained derivative is amphiphilic isosteviol-proline conjugated organo-catalyst (Figure 3E), which has high activity and stereoselectivity as compared to the proline [175]. The catalyst was utilized to encourage an aldol reaction. Chiral amphiphilic conjugate catalysts were outlined and integrated by covalently linking l-proline with an isosteviol, an aminoxylation of aldehydes, and ketones utilizing nitrosobenzene in phosphate buffer arrangement, bringing about the high level of enantioselectivities [176]. In another study, isosteviol amino catalyst conjugates as an organo-catalyst for asymmetric three-component Mannich Reactions. Isosteviol-proline conjugate (the proline derived organo-catalyst) was found to be a highly efficient catalyst for this reaction showing high stereoselectivity [177,178]. Isosteviol-amino acid conjugates were synthesized and used as chiral catalysts for the asymmetric three-component Mannich reaction with hydroxy acetone as a donor molecule. Good yields (up to 98%) and excellent stereoselectivities (up to 97:3 dr and 99% ee) were achieved in a short reaction time [179].

Isosteviol-derived bifunctional thiourea organo-catalysts were prepared using isosteviol as one of the scaffolds, and diamine as another scaffold [180]. The designed catalyst was proven to be effective in the adduct formation during the Michael reaction of acetylacetone and nitroolefins with high yield and good levels of enantioselectivity [180]. A similar result was experienced in the asymmetrical reaction of Enantioselective Michael Addition when isosteviol moiety was introduced contained in the thiourea catalyst [181]. This bifunctional thiourea catalysts explicitly catalyzed the reaction of aldehydes to maleimides in an excellent yield of 98% and enantioselectivities (up to 99%) [181].

### 5.2. Anti-Arsenic Contaminator

Arsenic contamination is a threatening concern and there is always a search for new chemicals that can reduce its poisonous ability. In this regard, ethanolic extract of *Pulsatilla nigricans* (EEPN) was chromatographed and was found to contain Dihydroxy-isosteviol-methyl-ester (DIME), which was tested for their ability to reduce arsenic toxicity. Around 20 mg/kg of sodium arsenate was tested on mice and their toxicity was examined for 30, 60, and 90 days. Mice models showed an increased level of reactive oxygen species (ROS), which is associated with arsenic contamination, leading to a decrease in sperm count, cellular damage, and other abnormalities. The arsenic-induced toxic effect in damaged cells and tissues of testis were reversed with EEPN [182].

## 6. Conclusions

Steviosides and their derivatives are biologically active compounds with its activity spectrum widely distributed such as antiviral, antibacterial, antioxidant, antitumor, analgesic, anti-inflammatory, antipyretic, and antifungal as well as playing other important roles. The present review is an effort to show the pharmacological importance of isosteviol compounds. Structure activity relation of some of the required isosteviol’s have also been reviewed. The stevioside is one of nature gifted compounds with both hydrophobic and hydrophilic properties. It provides opportunities to use its skeleton in the synthesis of a number of other beneficial derivative compounds and is also used to develop conjugated compounds with potent bioactivities. This information may help the medicinal chemists to design more selective, potentially active, and polyfunctional isosteviol analogues for the treatment of different diseases.
